# Factors associated with resilience among non-local medical workers sent to Wuhan, China during the COVID-19 outbreak

**DOI:** 10.1186/s12888-020-02821-8

**Published:** 2020-08-24

**Authors:** Jing Lin, Yun-Hong Ren, Hai-Jie Gan, Ying Chen, Ying-Fan Huang, Xue-Mei You

**Affiliations:** Nursing Department, Guangxi Medical University Affiliated Tumor Hospital, He Di Rd #71, Nanning, 530021 China

**Keywords:** COVID-19, Medical workers, Resilience, Coping style, Anxiety,depression

## Abstract

**Background:**

To investigate the resilience of non-local medical workers sent to support local medical workers in treating the outbreak of 2019 novel coronavirus disease (COVID-19).

**Methods:**

In February 2020, non-local medical workers who had been sent to Wuhan as support staff to respond to the COVID-19 outbreak were asked to complete an online survey composed of the Connor Davidson Resilience Scale (CD-RISC), Hospital Anxiety Depression Scale (HADS) and Simplified Coping Style Questionnaire (SCSQ).

**Results:**

Survey responses from 114 non-local medical workers were analyzed. CD-RISC scores were high (67.03 ± 13.22). The resilience level was highest for physicians (73.48 ± 11.49), followed by support staff, including health care assistants, technicians (67.78 ± 12.43) and nurses (64.86 ± 13.46). Respondents differed significantly in the levels of education, training/support provided by the respondent’s permanent hospital (where he or she normally works), and in their feelings of being adequately prepared and confident to complete tasks (*P* < 0.05). Resilience correlated negatively with anxiety (*r* = −.498, *P* < 0.01) and depression (*r* = −.471, *P* < 0.01) but positively with active coping styles (*r* = .733, *P* < 0.01). Multiple regression analysis showed that active coping (*β* = 1.314, *p* < 0.05), depression (*β* = −.806, *p* < 0.05), anxiety (*β* = − 1.091, *p* < 0.05), and training/support provided by the respondent’s permanent hospital (*β* = 3.510, *p* < 0.05) were significant associated with resilience.

**Conclusion:**

Our data show that active coping, depression, anxiety, and training/support provided by the respondent’s permanent hospital are associated with resilience. Managers of medical staff should use these data to develop psychosocial interventions aimed at reinforcing the resilience of medical workers during highly stressful and prolonged medical emergencies, as seen during the COVID-19 outbreak.

## Background

In December, 2019, the first case of 2019 novel coronavirus disease (COVID-19) was reported in Wuhan, a large city in Hubei province, China. The world turned its attention to Wuhan, and the high proportion of severe cases and the high mortality rate led to global panic [1, 2]. The World Health Organization raised the risk assessment of COVID-19 from “high” to “very high” at global level in the span of a month [3]. The rapid transmission of COVID-19 resulted in the diagnosis of more than 77,150 patients within 8 weeks [4]. The dramatic daily increase in the number of infected individuals placed a huge burden on medical workers. Doctors and nurses, in short supply, felt compelled to work longer hours without adequate rest.

In order to better control the epidemic and relieve pressure on local medical workers in Wuhan, the country’s top health authority organized medical workers from various provinces to be sent to the city. By mid-February 2020, 32,395 medical workers from around the country had been sent to Hubei province, and this number climbed to 42,600 by mid-March [5]. Their mission was to care for patients who had or were suspected of having infections, strengthen logistical support, help reduce pressure on local health-care personnel, and combat the ongoing novel coronavirus outbreak [6].

During an outbreak of infectious disease, medical workers are under great pressure due to heavy workload and hazardous working environment [7]. The severe situation causes mental health problems, such as anxiety and depression symptoms [8, 9]. These mental health problems affect the medical workers’ attention span, comprehension, and decision-making ability, which might hinder their ability to treat infectious disease and may also have lasting effects on their overall well-being [10, 11]. Medical workers in Wuhan surveyed in February 2020 showed anxiety prevalence of 59.6% and depression prevalence of 60% [12], suggesting the need for research and targeted interventions to ensure the mental health of medical workers on the COVID-19 front line [13].

These efforts may need to differentiate between Wuhan medical workers who normally live in the city and those from other areas of China who were sent there by the central government. In contrast to local medical workers in Wuhan, non-local workers must endure an unfamiliar work environment and new colleagues, and they must spend time away from their families under risky conditions. All these factors may act as stressors that take a greater toll on mental health than what local workers experience. On the other hand, many non-local personnel benefit from special training at their hospital of employment on how to prevent spread of infectious disease, and their families receive support from that hospital during the worker’s absence [14]. In addition, some non-local workers receive special attention from the host hospitals in Wuhan by virtue of their status as visiting workers [15]. Local workers, in contrast, may not receive these additional sources of support. These considerations raise the question of whether previously reported associations between work-related stress and mental health apply in the same way to non-local medical workers, but previous work on anxiety and depression among medical workers in Wuhan did not differentiate between locals and non-locals [12].

Resilience, which refers to the means and ability of effectively adjusting to or coping with adverse circumstances [16], may help maintain the health of individuals during a pandemic [17]. Indeed, the concept of resilience among medical workers facing natural disasters has attracted attention [13, 17]. Resilience is negatively associated with anxiety and depression symptoms of medical workers [18]. Therefore, we hypothesized that resilience would be positively associated with mental health among non-local health workers deployed to Wuhan during the COVID-19 pandemic.

The present study aimed to investigate the levels of resilience among non-local medical workers, and analyze the relationships of resilience to anxiety, depression and coping strategies. The study also examined which demographic factors may influence resilience levels among these workers. These findings may aid development of actionable psychosocial interventions to help increase the resilience of local and non-local health care workers during outbreaks of infectious diseases.

## Methods

### Design and sample

In February 2020, a cross-sectional on-line survey was conducted. Participants were medical workers currently working outside of Hubei province and sent to Wuhan to support first-line medical efforts against COVID-2019. Voluntary respondents were included if they held a professional certificate. Respondents were excluded if they reported experiencing a major negative event in the 3 months prior to going to Wuhan, such as personal health problems (i.e went to doctor or stayed in hospital), serious illness or death of a family member, divorce or separation, or involvement in a legal dispute.

### Data collection

During the outbreak of COVID-19, a private group chat comprised of non-local medical workers sent to Wuhan was created on the WeChat social media platform. These workers were identified through the authors’ professional and personal contacts. An on-line survey was constructed on an online questionnaire survey platform, and a QR code linking to the survey was generated and sent to the group. The contact information for a member of the research team was included in the survey in case subjects had any questions or required further explanation. Respondents provided written informed consent for their anonymized results to be analyzed and published for research purposes. The questionnaires took an average of 10 ± 4.48 min to complete.

### Demographic characteristics

Demographic information was collected through a self-designed questionnaire consisting of questions regarding personal variables, such as sex, age, occupation, marital status, education, training/support provided by the respondent’s permanent hospital(e.g.theoretical and operational training related to prevention of infectious diseases; taking cared of your families), adequate preparation, confidence to complete task.

### Hospital anxiety and depression scale

The Hospital Anxiety and Depression Scale (HADS) [19] was used to determine the presence of depression and anxiety among medical workers. It consists of 14 items, seven for depression and seven for anxiety. The score for each of these two subscales can range between 0 and 21. For each subscale, scores of 8–10 indicate mild symptoms, while scores of 11 or more indicate moderate/severe symptoms. Higher scores on each subscale reflect more severe symptoms. We removed the item “I feel as if I am slowed down” from the anxiety subscale, because it led to a lower Cronbach’s alpha. In this study, Cronbach’s alpha was 0.728 for anxiety and 0.702 for depression.

### Resilience

Resilience was measured using the Connor Davidson Resilience Scale (CD-RISC). This is a 25-item test that yields a score between 0 and 100. Higher scores indicate higher resilience. The 25 items are subdivided into three factors, including hardiness (13 items), strength (8 items), and optimism (4 items). The survey asks respondents to rate their agreement, using a 5-point Likert scale from “completely disagree” to “completely agree”, with statements such as “I am able to adapt when changes occur”, “I have one close and secure relationship”, “Sometimes fate or God helps me”, “I can deal with whatever comes my way”, and “Past successes give me confidence”. The Chinese version of the CD-RISC has shown good reliability and validity with adults [20]. In the present study, Cronbach’s alpha was 0.931.

### Coping style

Coping style was measured using the Chinese version of the Simplified Coping Style Questionnaire (SCSQ) [21]. It is a 20-item self-report questionnaire that includes two dimensions, active coping (12 items) and passive coping (8 items), with higher scores representing greater active/passive coping behaviors. Participants were asked to rate, using a 4-point Likert scale from 0 (“never”) to 3 (“very often”), how often they engaged in a particular behavior. Examples of active coping include distracting oneself through activities such as work, study or exercise, or speaking about one’s difficulties with others. Examples of passive coping include avoidance behaviors such as smoking, drinking, taking drugs, overeating or relying on others to solve the problem. Higher scores reflect more frequent use of the indicated coping style. In the present study, Cronbach’s alpha was 0.890 for the active coping dimension and 0.802 for the passive coping dimension.

### Statistical analyses

SPSS 20.0 was used for data analyses. Data showing a normal distribution were reported as mean ± SD. Differences in resilience scores between medical staff with different demographic characteristics were assessed for significance using the independent-samples *t* test and one-way ANOVA. Pearson correlation was used to explore the correlation of resilience with anxiety, depression, and coping style. Potential factors influencing resilience were analyzed using multiple linear regression. For all tests, the level of significance was set at *P* < 0.05.

## Results

### Sociodemographics

Of the116medical staff invited, 114 (98.3%) completed the survey. Participants were predominantly female (79.8%), and the most frequent age range was 31–40 years (43.0%). Far more respondents were nurses (61.40%) than doctors (18.4%), and the remaining 20.2% were other medical workers. Over half of participants (53.5%) were married, while 46.5% were single. Most medical staff (74.6%) held a bachelor’s degree as their highest degree (Table 1).

**Table 1:** Demographic characteristics and descriptive statistics of resilience in non-local medical workers sent to Wuhan during the COVID-19 outbreak (*n* = 114)

Characteristic	n (%)	Mean (SD)	F/t	P
Sex				
Male	23 (20.2)	69.17 ± 14.09	0.867	0.388
Female	91 (79.8)	66.49 ± 13.02		
Age (years)				
≤ 25	21 (18.4)	69.19 ± 11.50	1.446	0.233
26–30	32 (28.1)	69.31 ± 10.27		
31–40	49 (43.0)	64.08 ± 15.22		
41–50	12 (10.5)	69.25 ± 13.38		
Occupation				
Doctor	21 (18.4)	73.48 ± 11.49	3.640	0.029
Nurse	70 (61.4)	64.86 ± 13.46		
Support staff	23 (20.2)	67.78 ± 12.43		
Marital status				
Single	53 (46.5)	65.70 ± 12.98	−1.006	0.316
Married	61 (53.5)	68.20 ± 13.43		
Education				
Diploma degree	15 (13.2)	57.00 ± 13.78	5.660	0.005
Bachelor’s degree	85 (74.6)	68.94 ± 12.62		
Master’s degree or above	14 (12.3)	66.21 ± 12.04		
Training/support provided by the respondent’s permanent hospital				
Yes	42 (36.8)	71.43 ± 12.71	2.789	0.006
No	72 (63.2)	64.47 ± 12.92		
Adequate preparation				
Yes	81 (71.1)	69.13 ± 12.48	2.715	0.008
No	33 (28.9)	61.91 ± 13.78		
Confidence to complete task			2.557	0.012
Yes	109 (95.6)	67.70 ± 12.48		
No	5(4.4)	52.60 ± 21.56		

### Overall survey results

In general, participants showed a high level of resilience (67.04 ± 13.22). The active coping (26.61 ± 5.66) score was higher than the score of passive coping (10.32 ± 4.46). The medical staff had a mean score for anxiety of 7.40 ± 2.16 and depression of 5.40 ± 3.16.

### Factors associated with resilience

Non-local medical workers differed significantly in resilience depending on occupation, education, and mental health training, and depending on whether they felt adequately prepared and confident to complete tasks (Table 1). Nurses obtained a lower resilience score (64.86 ± 13.46) than doctors (67.78 ± 12.43) or other medical workers (73.48 ± 11.49). The resilience of medical workers with bachelor degrees (68.94 ± 12.62) was higher than that of workers with master’s degree or above (66.21 ± 12.04) or diploma degree (57.00 ± 13.78). These data suggest that higher resilience among non-local medical workers was associated with longer time spent on mental health training and preparation as well as greater confidence in being able to complete tasks.

### Correlational analysis

The Pearson correlation coefficients of all variables are shown in Table 2. Resilience, active coping, anxiety and depression significantly correlated with one another (*P* < 0.05). However, passive coping was not significantly correlated with resilience.

**Table 2:** Correlations among resilience, anxiety, depression and coping style in non-local medical workers (*n* = 114)

	Resilience	Active coping	Passive coping	Anxiety	Depression
Resilience	1				
Active coping	0.733^**^	1			
Passive coping	0.012	0.109	1		
Anxiety	−0.498^**^	−0.423^**^	−0.041	1	
Depression	−0.471^**^	−0.366^**^	0.270^**^	0.380^**^	1

^**^ *P*<0.05

### Multiple linear regression analysis

To identify factors that predict resilience, multiple linear regression was conducted with the following independent variables: occupation, education, mental health training, adequate preparation, confidence to complete tasks, anxiety, depression, active coping, and passive coping. Occupation and education had multiple categories, so they were treated as dummy variables. Stepwise linear regression was performed. Collinearity among the independent variables was not observed: the range of tolerance was 0.747–0.963 (evaluation criterion, > 0.1); variation inflation factor, 1.038–1.339 (evaluation criterion, < 10); and condition index, 1.00–23.204 (evaluation criterion, < 30).

The resulting model to predict resilience was significant (*F* = 45.244, *P* < 0.05) and showed an adjusted R^2^ of 0.610. In this model, resilience correlated positively with active coping (*β* = 1.314, *p* < 0.05) as well as with training and support (*β* = 3.510, *p* < 0.05), and negatively with depression (*β* = −.806, *p* < 0.05) and anxiety (*β* = − 1.091, *p* < 0.05; Table 3). Feelings of being prepared and confident to complete tasks were not significantly associated with resilience.

**Table 3:** Multiple linear regression analyses to identify factors that influence resilience

Model	Nonstandardized Coefficients		Standardized Coefficient	t	Sig.
	B	Std. Error	Beta		
Constant	50.189	7.091		7.007	0.000
Active coping	1.314	0.159	0.562	8.276	0.000
Depression	−0.806	0.274	0.193	−2.940	0.004
Anxiety	−1.091	0.412	−0.178	−2.647	0.009
Received training/support provided by the respondent’s permanent hospital	3.510	1.633	0.129	2.1493	0.034

*R* = 0.790; *R*^*2*^ = 0.624; Adjusted *R*^*2*^ = 0.610

Dependent Variable: resilience

## Discussion

Resilience is the ability to reduce the effects of a distressing event by anticipation and preparation, in other words to “bounce back” once it has occurred [16]. In this study, we analyzed resilience, anxiety, depression and coping strategies among non-local medical workers sent to Wuhan to support local staff in treating patients infected with COVID-19. The most important and significant finding of this study is that resilience was related to active coping, depression, anxiety, as well as training and support from the hospital. Resilience of non-local medical workers in this study was negatively associated with depression and anxiety. Our results suggest the potential usefulness of selecting more resilient medical workers to support the front line in major public health emergencies, and of creating resilience-building interventions to strengthen and preserve the mental health of medical staff working under challenging circumstances.

Our results suggest that resilience can protect a participant from feelings of anxiety and depression, which may help preserve mental health. Resilience helps medical workers recover better from trauma, which may help explain why resilient medical workers have lower anxiety and depression in the face of public health emergencies. Consistent with our study, analysis of more than 1000 nurses in Greece suggested that greater resilience was associated with lower anxiety [22]. In a study of Toronto-based health care workers who continued to work in hospitals one to 2 years after the SARS outbreak, incidence of new episodes of psychiatric disorders were similar to or lower than community incidence rates [23]. This may indicate a high level of resilience among these workers.

We also found a positive association between resilience and positive coping strategies. Consistent with our results, a survey of the psychological status of Chinese medical workers during the SARS outbreak in 2003 showed that they tended to adopt active forms of coping [24]. A study of US medical workers suggests that, during times of change in the workplace, encouraging nurses to use positive coping strategies may lead to higher levels of resilience [25]. A study in China suggested that new nurses with high levels of resilience could buffer the negative influence of workplace incivility by using a positive coping style [26]. Our data suggest that developing a training course on effective coping strategies would prove highly beneficial to improve individual’s resilience. For example, medical workers could be encouraged to share their troubles with colleagues and retain a sense of humor while working with patients and colleagues during a pandemic.

Our study found an inverse association of levels of anxiety and depression with level of training received from the hospital employment prior to dispatch to Wuhan. Similarly, a perception of being adequately trained and supported by the hospital showed an inverse association with level of psychological distress after the SARS outbreak [23, 27]. This suggests that hospital-provided training to workers can improve their resilience and help protect their mental health as they respond to a pandemic.

The respondents in this study had a mean CD-RISC score of 67.04 ± 13.22, scoring higher for resilience than Chinese medical workers analyzed in the non-epidemic period (59.50 ± 12.77) [28]. The higher score in our study may reflect a selection bias, since medical workers of higher resilience may have volunteered or been selected by their managers to take part in the challenging Wuhan mission. It is also possible that the higher resilience reflects the increased attention paid to mental health in China since the outbreak of SARS in 2003 and the Wenchuan earthquake in 2008 [29].

Nurses in our study scored lower on resilience than doctors or other support staff. Their score around 65 in our study is comparable to the score around 58 of nurses who survived the 2008 Wenchuan earthquake [30]. Nurses typically show higher psychiatric morbidity than administrative staff or doctors during pandemics [8, 31], which may lead to longer recovery time from stress. Research is needed to clarify to what extent this is due to less resilience and whether it can be prevented by appropriate interventions to boost resilience.

Feelings of being adequately prepared and confident to complete tasks was associated with resilience in bivariate analysis, but not regression analysis after controlling for covariates. Future research should investigate more samples to analyze whether adequate preparation and confidence in completing tasks affect the resilience of non-local health workers during a pandemic.

The health system could develop a training intervention that provides information about the status of the epidemic and infectious disease prevention to medical personnel before sending them to the front line, which may ease their fear and anxiety, thereby improving their resilience. Resilience may also be maintained by providing support(i.e financial, childcare, psychological counseling) to the families of front-line health workers. In addition, our study indicates that more time should be devoted to worker preparation, which could directly benefit a worker’s confidence to complete tasks. Due to the sudden nature of the outbreak, most supporting medical workers had very limited time from receipt of information to departure. In other words, our data show that the resilience of medical workers can be heavily influenced and maintained by appropriate organizational support strategies and pre-emptive planning.

### Limitations

Since questionnaires were filled out electronically at the participant’s convenience, we cannot exclude the possibility that factors in the survey environment (such as other people present) may have influenced participants’ responses. Moreover, due to time and place limitations, this study measured only end-point resilience. Future research should employ a longitudinal design to assess changes in resilience during a pandemic and how they may affect subsequent mental health. We were unable to assess effects of education level on resilience, anxiety or depression because nearly 75% of our respondents had bachelor’s degrees. Lastly, our study examined only non-local medical workers. Future studies should compare the mental health status and risk factors between local and non-local workers.

## Conclusions

To our knowledge, this is the first study to describe resilience levels of non-local medical workers sent to Wuhan during the COVID-19 outbreak, as well as associations between their resilience and mental health-related variables. Our findings suggest that efforts to enhance resilience should be tailored to the type of medical worker and his or her education level, provide training in knowledge and skills related to infectious diseases, and strengthen professional and personal assistance provided by the hospital. These strategies should help non-local medical workers fully prepare and feel confident when dealing with public health emergencies.

## Data Availability

All data generated during this study are included in this published article.

## Abbreviations

COVID-19: 2019 Novel Coronavirus Disease
CD-RISC: Connor Davidson Resilience Scale
HADS: Hospital Anxiety Depression Scale
SCSQ: Simplified Coping Style Questionnaire
